# Increased Systemic Malondialdehyde Levels and Decreased Mo/Co, Co/Fe^2+^ Ratios in Patients with Long-Term Dental Titanium Implants and Amalgams

**DOI:** 10.3390/jcm8010086

**Published:** 2019-01-12

**Authors:** María Eugenia Cabaña-Muñoz, José María Parmigiani-Izquierdo, Fabio Camacho Alonso, José Joaquín Merino

**Affiliations:** 1 Centro CIROM, Centro de Implantología y Rehabilitación Oral Multidisciplinaria, 30001 Murcia, Spain; mecjj@clinicacirom.com (M.E.C.-M.); jmparmi@clinicacirom.com (J.M.P.-I.); 2 Faculty of Medicine, University of Murcia, 30100 Murcia, Spain; fcamacho@um.es; 3 Hospital General Universitario Morales Meseguer, University of Murcia, 30100 Murcia, Spain

**Keywords:** toxicology of heavy metals, oligoelements, molybdenum (Mo), titanium (Ti), mercury, lipoperoxides (MDA), oxidative stress, free radicals and medicine, dental amalgams and titanium implants

## Abstract

Introduction: the biological safety of dental biomaterials has been questioned in human studies. Material and Methods: Several heavy metals/oligoelements were compared by Inductive Coupled Mass Spectrometry (ICP-MS) in hair samples from 130 patients (*n* = 54 patients with long-term titanium dental implants and amalgams (A + I group), 51 patients with long-term dental amalgam alone (A group), as well as controls (*n* = 25: without dental materials) of similar age. All patients (except controls) had had titanium dental implants and/or dental amalgams for at least 10 years (average: 17). We evaluated whether A + I patients could present higher systemic malondialdehyde levels (MDA) as compared to the A group. Results: The A + I group have lower molybdenum levels (A + I) and reduced Mo/Co and Mo/Fe^2+^ ratios, which could predispose them to oxidative stress by raising MDA levels as compared to the A group alone; our findings suggest that higher Co levels could enhance oxidative stress in the A + I group. However, there were no differences on metals from titanium alloy (Ti-6Al), Cr from crowns or Hg^2+^, Sn, Zn^2+^, Cu^2+^ levels between the A + I and A groups. Conclusion: patients with long-term dental titanium and amalgams have systemic oxidative stress due to rising MDA levels and lower Mo/Co and Mo/Fe^2+^ ratios than those with amalgams alone.

## 1. Introduction

Dental amalgam fillings contain 50% mercury, silver (Ag: 41%) together with minor constituents like tin (Sn: 8%), copper (Cu^2+^) or zinc (Zn^2+^) [1,2]. These heavy metals/oligoelements can be detected in biological samples (urine, plasma, hair) by Inductive Coupled Mass Spectrometry (ICP-MS) [3,4]. The safety of dental materials has been questioned in several clinical studies [5,6,7]. Different oligoelements like Cu^2+^, Fe^2+^, Mn^2+^, Zn^2+^, or molybdenum (Mo) are necessary cofactors for certain enzymatic antioxidant activities [8]. For instance, the oligoelement Mo is required for the formation of the unique protein called aminodoxine (mARC), which is involved in mitochondrial function. Mo is also a cofactor of xantin oxidase, which transforms hypoxanthine and xanthine into uric acid and can thus act as an antioxidant [9]. Deficiencies of vanadium (V) or Mo have not been detected in healthy people. The National Academy of Science (USA) estimated day Mo intake as 106 micrograms for adult men and 76 mg for adult women. Other minerals like iron are part of enzymes as such cytochrome, catalase or hemoglobin/myoglobin (oxygen carriers) and contribute to bone synthesis, joints, and cartilage formation. The vanadium (V) oligoelement is a cofactor in blood sugar metabolism, lipid and cholesterol metabolism, bone and teeth development (For a review on oligoelement function see Soetan et al., 2010 [10]). Cobalt (Co) is necessary for B12 vitamin synthesis and also stimulates the formation of blood cells. A Co overdose is toxic and can cause heart problems as well as lead to red cell overproduction. Calcium (Ca^2+^) is a necessary biol trace element for many enzymatic reactions and its deficit can provoke metabolic alterations and dentition problems in children and adults [10,11]. The function of oligoelements in odontology is still unknown/little studied.

Nowadays, the replacement of dental amalgams is justified by the use of more biocompatible dental materials or aesthetic treatments in patients. These new dental biomaterials must be compatible, bisphenol-A free composites, coating materials: crowns, bioceramics, zirconium implants, cements from glass ionomer and zinc phosphate, etc. Consequently, clinical protocols to prevent vapor mercury release during dental amalgam removal are necessary [12]. However, these treatments could induce some kind of toxicity should galvanic heavy metal release occur in the mouth over time as a consequence of an interaction between heavy metals. Galvanism is a corrosion phenomenon that may lead to ion metal release from dental alloys into distal organs [13,14,15]. These prosthetic dental materials contain nanometals (Co, Cr, Au, Ag, Ti), resins (Si), and ceramics [13,14,15,16], that could provoque detrimental effects in patients [17]. These biomaterials could also provoke metabolic alterations or lead to oligoelement/s deficit in patients with long-term dental biomaterials. Titanium alloy (Ti-6Al-4V) is biologically inert but TiO_2_ nanoparticles toxicity has been reported “in vitro” [18].

### 1.1. Aim

The main aim was to evaluate whether patients who have long-term dental titanium implants and amalgams have systemic oxidative stress due to rising malondialdehyde (MDA) levels and lower Mo/Hg^2+^, Mo/Co, Mo/Fe^2+^ ratios than those with long-term dental amalgams alone.

### 1.2. Specific Aims

We evaluated levels of heavy metals (Hg^2+^, Al, Ba, Sn, Ti, Sb, As, Cd, Pb, Pt, Tl, Th, U) and oligoelement (Mo, Ca^2+^, V, Mn^2+^, B, I, Sr, S, Fe^2+^, Ge, P, Zn^2+^, Cu^2+^, Cr, Co, Ni, Co) in patients with long-term dental titanium and amalgams (A + I) as compared to those with long-term dental amalgams alone (A) and to controls (Cont: without dental fillings or titanium implants).

We evaluated whether patients with long-term titanium dental implants and amalgams (A + I) could be more susceptible to oxidative stress and rising systemic malondialdehyde (MDA) levels than patients with long-term amalgams alone (A).

Once, we detected lower molybdenum (Mo) levels in patients with long-term titanium implants and amalgams (A + I group), we then analyzed their Mo/Hg^2+^, Mo/Co, Mo/Fe^2+^ ratios and systemic taurine levels as compared to those with long-term dental amalgams alone and the control subjects.

## 2. Materials and Methods

This study was conducted following the Declaration of Helsinki (1974 and revised form, 2000). All Subjects were properly informed and consented to participate by signing the appropriate informed consent forms (CIROM Clinic, Murcia, Spain). In addition, all efforts were made to protect patient privacy and anonymity. The CIROM Center has been approved and certified by AENOR Spain (Spain; CIROM Certificate for dental services; 2014/0601/CD/01 number; 2014/0601/ER/01 following UNE-EN ISO 9001: 2015 and UNE-UNE 179001-2001 Directive, Murcia, Spain). All patients enrolled in the present study fulfilled the following criteria.

### 2.1. Inclusion Criteria: Size Sample

We quantified several heavy metals/oligoelements by ICP-MS in 130 hair samples from patients (60% of them women). Patients were classified into participants who had had dental titanium implants and amalgams for at least 10 years (average: 17 years in mouth, A + I group, *n* = 54) and those with long-term dental amalgams alone (A, *n* = 51) together with 25 age-matched controls of similar age (Cont: without implants and/or dental amalgams). Patients with long-term dental titanium implants alone are not often encountered in a dental clinic since most previously had long-term dental amalgam fillings. Thus, participants with titanium alone are not included in the present study.

Heavy metals/oligoelements were quantified by ICP-MS—Inductively coupled plasma-mass spectrometry—In hair samples (µg/g hair). Mercury and silver are 50% (Hg^2+^) and 40% (Ag), respectively of total dental amalgam weight; the oligoelements (Sn, Cu^2+^, Zn^2+^) are 8% of total weight and titanium (Ti), aluminum (Al), and vanadium (V) are constituents of titanium dental alloy (Ti-4Al-6V) [16]. The age of patients with long-term dental titanium implants and amalgams (A + I) was 58 years old and participants with long-term dental amalgams 49 years old. Controls were 42 years old. Hair samples were collected close to the scalp (0.25 g from the occipital area) and many heavy metals and/or oligoelements were compared in hair samples from study groups by ICP-MS (Dr. DATA USA); this is a pioneer laboratory with over 35 years of experience in ICP-MS analysis. All heavy metal/oligoelements were expressed as µg/g of hair (Hg^2+^, Cu^2+^, Zn^2+^, Sn, Ti, Al, V, Ni, Cr, Co, Mo, Ba, Ca^2+^, Sr, Mn^2+^, Fe^2+^, I, Ge, P, S, Cd, Pb, Sb, As, Pt, Tl, Th, U).

The average number of titanium dental implants were four (A + I: minimum: 1, maximum: 7) in those patients with long-term implants and amalgams and were four in patients with long-term dental amalgams alone (A: minimum: 1, maximum: 7). The dental biomaterials had been present in their mouth for at least 10 years (average: 17 years). Control subjects without dental biomaterial were also included in this design. All recruited patients have a medium/high sociocultural status and live in Spain (80% are from Murcia and 20% from Alicante). The percentage of women was 60%.

Systemic malondialdehyde (MDA) levels were measured as an index of lipoperoxidation by thiobarbituric acid assay (TBARS) and systemic taurine levels were assayed by using High-Performance Liquid Chromatography (HPLC) [19] and compared between all groups; taurine is a sulfur amino acid that also contributes to heavy metal detoxification.

### 2.2. Exclusion Criteria

Patients who had periodontal disease or those with metabolic diseases/diabetes, neurological/psychiatric disorders (4th Edition, The Diagnostic and Statistical Manual of Mental Disorders (DSM) IV) were excluded. Patients taking chelators or other chronic medications (stimulants, anticonvulsants, antidepressants or psychiatric/bipolar drugs) were not accepted in the present study. In addition, patients who had history of metabolic disease (liver, kidney disease, lupus) were also excluded. Additionally, patient tattoos were excluded in order to avoid interferences in mercury levels in the present study.

### 2.3. Thiobarbituric Acid Assay (TBARS): Malondialdehyde Levels (MDA) as an Index of Lipoperoxidation

Malondialdehyde (MDA) levels were quantified following a protocol from Tiwari and Chopra. 2011 [20]. During the lipoperoxidation process, the 4-hydroxinonenal formation could induce arachidonic acid oxidation, which reacts with proteins. The standard curve was created by a gradient of TBARS concentration and the TBARS reactive (thiobarbituric acid) was heated at 100 °C; 0.5 mL of TBARS was added to the upper fraction of plasmas. Briefly, the heated reactive was added to the plate in TBA (pH 6.8) and 0.5 mL Tris-HCl buffer. The plasma samples were isolated from blood by centrifugation at 1300 rpm for 10 min; These samples were incubated at 37 °C for 2 h at room temperature (R.T.) before adding 1 mL of TBA (0.67% for 10 min). As soon as all samples were cold, 0.5 mL distilled water was added to all 96 wells and the absorbance measured at 532 nm [20]. The TBA content was quantified by interpolation within the standard curve and expressed as mg of MDA (MDA/mg protein); finally, all data were expressed as a percentage of the control group, which is 100%. Controls were subjects without dental biomaterials (titanium implants or amalgams).

### 2.4. Statistical Analysis

All data were quantified by SigmaPlot (11.0) (Systat Software, Inc., San Jose, CA, USA) and SPSS 15.0 (IBM Corporation, Armonk, NY, USA) software. Means and standard deviation were estimated for all evaluated parameters. The table and graphs show the mean value for each heavy metal/oligoelement plus standard error media (S.E.M); the S.E.M equals the standard deviation divided by the square root of the sample size. Non parametric Kruskal Wallis analysis was performed for cases of non-homogeneity of variance if the Levene test was statistically significant (*p* < 0.05). The post hoc Mann Whitney or Dunn’s analysis are non parametric tests used for multiple comparisons among groups; the Dunn’s task is applied for unequal sample size for comparisons between groups and controls subjects, respectively. The ANOVA (Analysis of variance) identifies possible significant differences between groups in case of homogeneity of variance when the Levene test is not significant (*p* > 0.05). In this case, the post hoc Bonferroni test analyzed multiple comparisons between groups. Differences were statistically significant if *p* < 0.05 and highly significant when *p* < 0.01. The correlations between data were evaluated by r Pearson or Spearman test depending on the homogeneity of the data.

## 3. Results

### 3.1. Comparative Heavy Metals/Oligoelement Levels in Patients with Long-Term Dental Titanium Implants and/or Amalgams Alone

**Figure 1:**
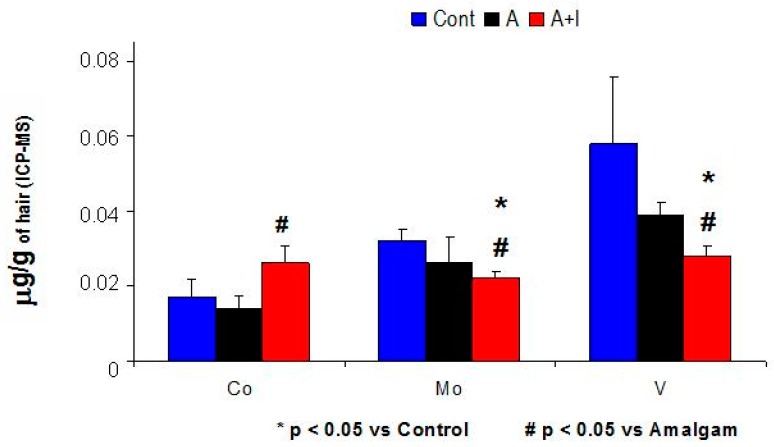
Increased cobalt (Co) and lower molybdenum (Mo) and Vanadium (V) levels in patients with long-term dental titanium implants and amalgams (A + I) than those with long-term dental amalgams alone (A). Cont, Control.

Patients who had long-term dental titanium implant and amalgams (A + I) showed higher cobalt (Co) and nickel (Ni) levels in hair as compared to patients with long-term dental amalgam fillings alone (A, *p* < 0.05 in both cases). However, the Hg^2+^, Zn^2+^, Cu^2+^ levels from dental amalgams, crowns (chromium: Cr) or titanium implants (Ti, Al) did not significantly differ among patients with long-term dental implants and amalgams (A + I) as compared with those with long-term dental amalgams alone (A, *p* > 0.05, n.s. (not statistically significant), Table 1 and Figure 1).

In addition, increased Hg^2+^, Al, Zn^2+^, and Ni levels were observed in patients with long-term dental titanium implants and amalgams than in their respective controls (without dental biomaterials, Table 1).

Table 1: Patients who had long-term dental titanium implants and amalgams (A + I) had higher mercury (Hg^2+^), aluminum (Al), zinc (Zn^2+^) as compared to controls.

Patients who had long-term titanium implants and dental amalgams (A + I, *n* = 54) had lower nickel (Ni), and cobalt (Co) levels in hair that those with long-term dental amalgams alone (*n* = 51) or also controls (*n* = 25, without dental biomaterials). Data are expressed as mean heavy metal plus relative error media (S.E.M: standard deviation/n root). The asterisk shows statistical significance (* *p* < 0.05 vs. control; ^#^ *p* < 0.05 vs. dental amalgam group, n is the sample size); n.s.: not statistically significant (*p* > 0.05). S.E.M: Standard Error median. Cont: Controls (patients without titanium dental implants or dental amalgams, *n* = 25). A: Patients with at least one dental amalgam, *n* = 51. A + I: Patients who have long-term titanium dental implant and amalgams, *n* = 54. n (total): 130 patients. n.s. means *p* > 0.05. H is the Kruskal Wallis value and F is the data for ANOVA (Analysis of variance).

### 3.2. Molybdenum (Mo) and Vanadium (V) Levels Were Significantly Lower in Patients with Long-Term Dental Amalgam and Titanium Implants (A + I) Than in Those with Long-Term Dental Amalgams Alone (A) or Controls

Patients who have long-term dental titanium implants and amalgams (A + I) had lower vanadium (V) and molybdenum (Mo) levels in hair than those with long-term dental amalgams alone (A, *p* < 0.05 in both cases, Table 1 and Figure 1); The (A + I) group had lower vanadium (V) and molybdenum (Mo) levels than their respective controls (*p* < 0.05). However, patients with long-term dental amalgams alone (A) had levels similar to those of controls (without dental biomaterials, n.s.). All results are expressed as mean of µg/g of hair.

### 3.3. Iron (Fe^2+^): Patients Who Have Long-Term Amalgams and Titanium Dental Implants (A + I) Have Significantly Higher Iron Levels than Those in with Long-Term Dental Amalgams Alone (A)

The Kruskal Wallis test revealed a significant rise in iron levels (Fe^2+^:H = 5.83; *p* = 0.05). The Mann Whitney test showed higher iron levels among patients with long-term titanium and dental amalgams (A + I) than in those with long-term dental amalgams (A, *p* < 0.05, Table 2).

### 3.4. Low Molybdenum (Mo) Levels as well as Lower Mo/Co and Mo/Fe^2+^ Ratios in Patients Who Have Long-Term Dental Titanium Implants and Amalgams (A + I) than in Those with Long-Term Dental Amalgams Alone (A)

The ANOVA showed a greater molybdenum (Mo) deficit among patients who had long-term dental titanium implants and amalgams (A + I) than in those with long-term dental amalgams alone (A) (H = 9.14, *p* = 0.001). The post hoc Dunn’s test confirmed greater Mo reduction among patients with titanium dental implants (A + I) and controls (*p* < 0.05). However, Dunn’s test did not find a significant effect among those with long-term dental amalgams alone (A) and controls (*p* > 0.05, n.s.).

**Figure 2:**
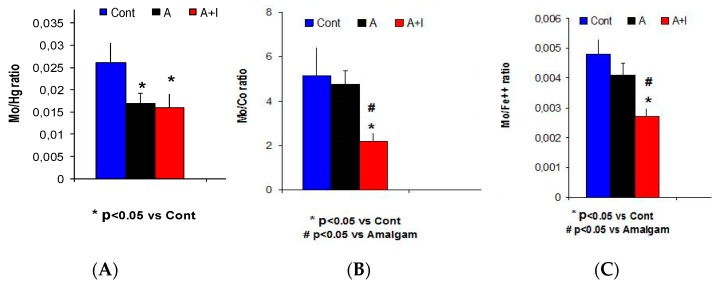
The Mo/Co and Mo/Fe^2+^ ratios were significantly decreased in the A + I group as compared to those with long-term dental amalgams alone A (**A**) The Mo/Hg^2+^ ratio is significantly reduced in both experimental groups with dental biomaterials (A + I or A) as compared to controls. The Mo/Co ratio (**B**) as well as Mo/Fe^2+^ ratio (**C**) were significantly decreased in A + I patients as compared to those with long-term dental amalgams alone and controls (Cont).

There were lower Mo/Co and decreased Mo/Fe^2+^ ratios in patients with long-term dental titanium implants and amalgams (A + I) than in those with long-term dental amalgams alone (Mo/Hg^2+^: H = 11.9, *p* = 0.003; Mo/Co: H = 10.89, *p* = 0.004; Mo/Fe^2+^: H = 15.13; *p* < 0.001). The striking correlation between Mo/Hg^2+^ and Mo/Co ratios (*r* = 0.45, *p* = 0.005, *n* = 54) observed in the present study highlights the relevance of our findings; Dunn’s analysis revealed lower ratios among patients with long-term dental implants and amalgams (A + I) than in those with long-term dental amalgams alone (A) or the control subjects (*p* < 0.05). However, these ratios did not differ between patients with long-term amalgams alone (A) and controls (*p* > 0.05, n.s., Table 2 and Figure 2).

### 3.5. Systemic Malondialdehyde (MDA) Rises among Patients with Long-Term Dental Titanium Implants (A + I) as Compared to Those with Long-Term Dental Amalgams Alone (A)

**Figure 3:**
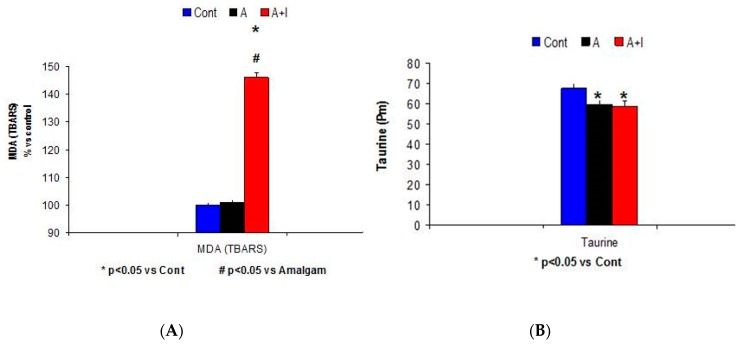
Higher systemic malondialdehyde (MDA) levels (**A**) and lower systemic taurine (pM) levels (**B**) in patients with long-term amalgams and dental titanium implants (A + I) and amalgams (A) alone.

The Kruskal Wallis analysis revealed a systemic malondialdehyde (MDA) rise among patients who had long-term titanium implants and amalgams (A + I) as compared to participants with long-term dental amalgams alone (A) and control subjects (Cont) (H = 33.6, *p* < 0.001. Figure 3A).

#### Taurine Levels

There was a very significant effect for systemic taurine levels F (2.113) = 5.02, *p* = 0.01. Systemic taurine levels were significantly reduced in patients with long-term titanium dental implants and/or long-term dental amalgams as compared to controls, respectively (*p* < 0.05 in both cases); there was no effect in the A + I group as compared to those with long-term dental amalgams alone (A, *p* > 0.05, n.s., Figure 3B).

### 3.6. Oligoelement Levels Found in Patients with Long-Term Titanium Implant (A + I) and/or Dental Amalgams alone (A) (Ca^2+^, Sr)

#### 3.6.1. Calcium (Ca^2+^)

The Kruskal Wallis analysis revealed a significant effect on calcium (Ca^2+^) level (H = 8.52, *p* = 0.014). The Dunn’s or Mann Whitney post hoc analysis showed higher Ca^2+^ levels among patients with long-term dental implants and amalgams than among control subjects (without implants or dental amalgams, *p* < 0.05). In addition, Ca^2+^ levels detected in the A + I group tended to rise as compared with patients with long-term dental amalgams alone (A, *p* = 0.12, n.s.) or controls, respectively (*p* = 0.11, n.s.; see Table 3).

#### 3.6.2. Strontium (Sr)

The Kruskal Wallis revealed a significant effect on Sr levels in hair (H = 6.096, *p* = 0.047). The Mann Whitney post hoc test showed higher levels in patients with long-term dental titanium implants and amalgams (A + I) than in control subjects (*p* < 0.05). Sr levels were higher in patients with dental amalgams alone than in controls, but not significantly so (*p* = 0.11, n.s., Table 3).

#### 3.6.3. Sulfur (S)

The ANOVA data showed a trend to lower S levels (F (2.72) = 2.67, *p* = 0.076, n.s.). The Bonferroni post hoc analysis revealed a significant reduction in S levels among patients with long-term dental amalgams as compared with control subjects (without dental biomaterials, *p* < 0.05). In addition, patients with long-term dental titanium implants and amalgams (A + I) tended to show lower S levels than those with long-term dental amalgams alone (*p* = 0.092; n.s., Table 3).

### 3.7. Metals of Environmental Exposure

#### 3.7.1. Barium (Ba): patients with Long-Term Titanium Dental Implant and Amalgams Have Significantly Higher Ba Levels than Those with Long-Term Dental Amalgams Alone

The Kruskal Wallis showed significantly higher barium (Ba) levels (H = 15.77, *p* = 0.001). The Mann Whitney test showed higher Ba levels in both patient groups (implant and/or amalgam) than controls (*p* < 0.05). In addition, patients who had long-term titanium implants and dental amalgams had (A + I) significantly higher Ba levels than those with long-term amalgams alone (A, *p* < 0.05, Table 4).

#### 3.7.2. Cadmium (Cd), and Lead (Pb) Levels

The Kruskal Wallis analysis did not show a significant effect on cadmium (Cd) or lead (Pb) levels ((H = 2.97, *p* = 0.22: n.s. for Pb) or (H = 3.43, *p* = 0.18, n.s. for Cd)). However, there was a trend towards higher levels for both heavy metals among patients who have long-term titanium implants (A + I) than in patients with long-term dental amalgams alone (*p* = 0.11, n.s. for Pb or *p* = 0.064 for Cd, n.s.). Table 4 shows these data.

Finally, there was no effect on several oligoelements (Mn^2+^, Ge, I, P, Table 3) among study groups in the present study (Sb, As, Pt, Tl Th, U, Table 4).

### 3.8. Correlations between Certain Oligoelements (Ca^2+^, Mo, Fe^2+^, Co, V, Cr, Co) and Heavy Metal Levels (Hg^2+^) in Patients with Long-Term Titanium Implants and Dental Amalgams (A + I Group)

The *r* Pearson/Spearman analysis indicated a correlation between molybdenum (Mo) and certain oligoelement levels (Ca^2+^, Mg^2+^, Fe^2+^) in patients with long-term titanium implants and dental amalgams (A + I group). The r Pearson or Spearman test revealed a correlation between Mo/Hg^2+^ and Mo/Co ratios as well as between Mo/Hg^2+^ and Mo/Fe^2+^ ratios. The Mo levels correlated with Ca^++^ levels in A + I participants; In addition, we observed a correlation between vanadium (V)-chromium (Cr) levels, as well between S and Zn^++^ levels in the A + I group. All results were expressed as mean of µg/g of hair (Table 5).

## 4. Discussion

### The Rise in Cobalt (Co) and Nickel (Ni) in Hair of Patients with Long-Term Titanium Implants and Amalgam Fillings as Compared to Patients with Long-Term Amalgams Only

The cobalt (Co) and Ni rose in our patients with long-term titanium dental implants and amalgams (A + I) in comparison to patients who had long-term amalgams alone (A). This study recruited participants who had had dental titanium implants (A + I) and/or dental fillings for at least 10 years (average: 17 years) because heavy metals may produce detrimental effects more than 5–7 years after initial exposure [1,17].

Titanium (Ti) is the major component in dental titanium alloys, while Cr, Co, and Ni are coating materials for crowns [13]. Surface treatments of Ti implants can also enhance metal reactivity of their surface, possibly increasing metal ion release [14]. However, Ti, Al, or Cr levels did not significantly differ between patients with the dental implants and amalgams (A + I) and those with long-term dental amalgams alone (A); this observation suggests that Ti-6Al-4V alloy is biocompatible given the 100% of implant success in the present study. The absence of effects on Ti levels in the present study agrees with findings from a clinical study with 67 patients with a hip arthroplasty. In fact, there was no effect on systemic titanium (Ti) levels as compared with controls in that study [21]. However, the titanium exposure time is longer in patients with a hip arthroplasty than in patients with dental titanium implants and amalgams. Factors such as implant stability, mechanical stress, and galvanism may enhance ion metal release [16,22] when dental amalgam fillings are close to titanium alloys. The rises in Co and Ni levels could reflect galvanic ion release over time in patients with long-term dental implants and amalgams (A + I). These findings agree with the data of Camacho-Alonso et al. on saliva samples [23]. The rise in mercury (Hg), aluminum (Al) and barium (Ba) levels in the A + I group suggest that galvanism could lead to ion release [15,24]. However, the contribution of titanium dental implants to galvanic corrosion of dental amalgam seems to be minimal because successful dental implants are only in contact with bone and not exposed to the oral cavity; however, that could be enough to affect Mo/Co or Mo/Fe^2+^ ratios and also lead to raised Co levels as well as increased iron (Fe^2+^) levels among A + I patients as compared to those patients with long-term dental amalgams alone (A).

These dental amalgams contain mercury (50% of total dental amalgam weight) [1]. Other heavy metals such as barium (Ba) have been found to be higher by dentists [2]. Ba was also higher in patients with long-term dental implant and amalgams than in control subjects. In addition, Hg^2+^ and Al were higher in patients with titanium dental implants and amalgams (A + I) as compared with their respective control subjects but not compared to those with long-term dental amalgams (A). It is known that blood and Hg^2+^ urine levels only identify acute exposure to mercury but underestimate total mercury retention within tissues and organs [25]. For this reason, hair levels of metals/oligoelements were quantified by ICP-MS following previous studies because ICP-MS analysis is very accurate for chronic heavy metal detection in patients [26,27,28,29,30]. Our results do not exclude the possibility that mercury may be accumulating within tissues/organs such as kidneys [31]. Their total mercury/and aluminum levels in hair detected by ICP-MS did not differe between patients who had long-term implants (A + I) and those with long-term dental amalgams alone (A) [4]. The USA-NHANES: National Health and Nutrition Examination Survey, study reported that mercury in hair but not in urine correlated with antinuclear antibody ANA levels (Antinuclear Antibody: a lupus marker) in 1352 women of reproductive age [28].

The Mo levels are lower among A + I patients than those with long-term dental amalgams alone (A). The oligoelement, Mo, regulates the redox balance and also certain enzymatic activities involved in xenobiotic detoxification; Mo is a cofactor in xanthine-oxidase enzymatic activity (it forms acid in the presence of Mo) [9] and healthy amounts of uric acid (its final product) play an antioxidant role [32]. Since Mo decreased among A + I participants and their cobalt (Co) levels rose, we cannot exclude the possibility that systemic malondialdehyde (MDA) levels could indirectly reflect enhanced susceptibility to oxidative stress among A + I patients. In addition, Co induces inflammatory responses via TLR-4 activation in culture cells, responses which are abolished by blocking TLR-4 receptors [33]. In agreement with this hypothesis, we detected lower Mo/Co and Mo/Fe^2+^ ratios in the (A + I) group than in those with long-term dental amalgams only (A); The low Mo/Co ratio (A + I) suggests a Mo deficit in patients with long-term implants and amalgams (A + I) although Mo levels were similar to controls in patients with long-term amalgams alone. These lower ratios could contribute to the greater oxidative stress in patients with long-term dental titanium implants and amalgams than in those with long-term dental amalgams alone. However, the risk of molybdenum deficiency is quite low in humans (USA) [10] and it is not possible to establish a direct causal relationship between Mo deficits and systemic MDA elevations in the A + I group. Mo is a cofactor for the xanthine-oxidase enzyme, which releases iron from ferritin in the intestinal mucosa, liver, and in placenta in its ferrous form [34,35,36]. The direct relationship between Mo deficits and iron accumulation is a possibility in the A + I group. Since enzymatic molybdenum (Mo)-dependent activities require iron [37], these low Mo levels observed in conjunction with negative Mo–Fe^2+^ and Mo–Ca^++^ correlations could suggest iron accumulated in the tissues of the A + I group. Iron is important for Mo dependent enzymatic activities, cytochromes, and electron transport [10] and it is a cofactor for aldehyde oxidase enzymatic activity, which is critical during the first phase (Phase I) of liver detoxification [9]. We have not yet seen evidence on symptoms associated with Mo or V deficiency in healthy humans with long-term dental biomaterials in the present study [10]; As these enzymatic activities are dependent on Mo levels, we suspect that Mo deficits could lead to iron (Fe^2+^) accumulation among A + I patients.

Mo is also necessary for sulfite oxidase to convert sulfite to sulfate [9] and sulfur (S) is an oligoelement that is present in sulfur amino acids [i.e., taurine], which contribute to detoxificate xenobiotics, including heavy metals [38]. These low systemic taurine levels (sulfur aminoacid) observed in our patients may indirectly reflect poor detoxification capacities [39] in those patients with long-term dental titanium implants and/or amalgams. Conversely, we previously reported chronic compensatory responses against heavy metal release in women who show higher superoxide dismutase-1 (SOD-1) activity than control subjects without dental biomaterials [4]. We observed that systemic taurine levels are low in patients with long-term dental amalgams and they also have decreased Mo/Hg^2+^ ratios (A group) and Ba iron accumulation as compared with their respective controls. However, patients with long-term dental implants and amalgams could be more sensitive to emergent disease or cardiovascular problems in the future.

Finally, certain oligoelements (Ca^++^, Mn^++^, P, Fe, Ge, As, Sb, Pt, Tl, Th, U) and heavy metal levels did not differ between patients with long-term titanium implants and amalgams (A + I group) and those with amalgams alone (A). Further clinical trials should confirm whether molybdenum (Mo) supplementation can reduce oxidative stress and prevent heavy metal accumulation in patients with long-term dental amalgams and titanium implants (A + I group). Metals of environmental exposure like Cd tend to be raised, possibly as a consequence of defective antioxidant responses among A + I patients. In addition, Cd detected levels from plants in Murcia area (Spain) exceeded the European allowed limit [40].

As a general rule, safe clinical protocols for dental amalgam replacement by composites [12] as a precautionary principle must be applied in the odontologic field [41]. The exposure to electromagnetic fields can affect mercury release and be a potential hazard for hypersensitive people and pregnant women [42]. Finally, these clinical findings suggest that titanium plates and screws should be removed once bone consolidation occurs in the time after a conventional orthognathic surgical procedure.

## 5. Conclusions

The Mo/Hg^2+^ and Mo/Co ratios decreased in patients who had long-term dental amalgams and titanium implants (A + I), suggesting reduced molybdenum (Mo) and vanadium (V) levels. As the malondialdehyde (MDA: an index of lipoperoxidation) and cobalt (Co) are higher in these patients, we suspect they are more susceptible to oxidative stress than those with dental amalgams alone (A). The nickel (Ni) and cobalt (Co) elevations derived from crowns (which cover the titanium implants) suggest a minimal galvanic release. In addition, mercury (Hg^2+^), aluminum (Al) or titanium (Ti) levels as well as certain oligoelement levels did not differ between the A + I patients and A patients alone (A). The possibility that Mo deficiency and Co elevations could lead to iron (Fe^2+^) accumulation agrees with enhanced oxidative stress responses derived from the higher MDA levels and reduced taurine levels seen in patients with long-term dental titanium implants and amalgams. However, patients with long-term dental amalgams (A) alone seem to develop a compensatory mechanism against ion metal release as compared to controls (without dental biomaterials).

## Figures and Tables

**Table 1:** Levels of heavy metals from amalgams (A), titanium dental implants and crowns (cobalt (Co), nickel (Ni) and chromium.

Metal (µg/g Hair)	Control (Cont)	Amalgams (A)	Amalgams+ Implants (A + I)	*p*-Value (H or F)
Hg^2+^	1.28 ± 0.51	2.72 ± 0.24 *	2.67 ± 0.29 *	H = 8.4, *p* = 0.015
Sn	0.3 ± 0.18	0.9 ± 0.32	0.57 ± 0.19	H = 1.84, *p* = 0.44; n.s.
Cu^2+^	29.1 ± 7.4	41.8 ± 12	29.1 ± 5.7	H = 3.93, *p* = 0.14; n.s.
Zn^2+^	185 ± 9.3	211 ± 12.8 *	217 ± 7.4 *	H = 5.8, *p* = 0.05
Ti	0.44 ± 0.037	0.47 ± 0.07	0.48 ± 0.065	H = 1.02 *p* = 0.6; n.s.
Al	2.08 ± 0.1	4.88 ± 0.82 *	3.37 ± 0.4 *	F = 14.5, *p* < 0.001
V	0.058 ± 0.018	0.039 ± 0.0033 *	0.028 ± 0.003 *^,#^	H = 11.63, *p* = 0.003
Ni	0.13 ± 0.06	0.159 ± 0.04	0.39 ± 0.18 *^,#^	H = 6.6, *p* = 0.037
Co	0.017 ± 0.0045	0.014 ± 0.0034	0.026 ± 0.0049 ^#^	H = 13.9, *p* < 0.001
Cr	0.35 ± 0.13	0.35 ± 0.013	0.35 ± 0.01	F = 0.12, *p* = 0.88; n.s.

* *p* < 0.05 vs. Control ^#^ *p* < 0.05 vs. Amalgam. H, the Kruskal Wallis value; F, the data for ANOVA.

**Table 2:** Mean values for Mo, Fe^2+^, and Mo/Fe^2+^, Mo/Co ratios between groups (µg/g hair).

Metal (µg/g Hair)	Control (Cont)	Amalgams (A)	Implants + Amalgams (A + I)	*p*
Mo	0.032 ± 0.0033	0.026 ± 0.007	0.024 ± 0.002 *^,#^	H = 9.14, *p* = 0.01
Fe^2+^	7.73 ± 0.58	7.42 ± 0.43	8.89 ± 0.5 ^#^	H = 5.83, *p* = 0.05
Mo/Hg^2+^	0.026 ± 0.0045	0.017 ± 0.0022 *	0.016 ± 0.003 *	H = 11.9, *p* = 0.003
Mo/Co	5.13 ± 1.27	4.77 ± 0.59	2.17 ± 0.4 *^,#^	H = 10.89, *p* = 0.004
Mo/Fe^2+^	0.048 ± 0.037	0.042 ± 0.0017	0.015 ± 0.002 *^,#^	H = 15.13, *p* < 0.001

* *p* < 0.05 vs. Control ^#^ *p* < 0.05 vs. Amalgam.

**Table 3:** Mean oligoelements ± standard error media (S.E.M) levels in hair for the study groups (Ca^2+^, Sr, S, Mn^2+^, Ge, I, P).

Metal (µg/g Hair)	Control (Cont)	Amalgams (A)	Implants + Amalgams (A + I)	*p*-Value
Ca^2+^	722 ± 142	1096 ± 191	1695 ± 233 *^, #^	H = 9.51, *p* = 0.009
Sr	8.7 ± 3.7	12 ± 3.3	17 ± 2.88 *	H = 6.09, *p* = 0.047
S	48088 ± 426	47151 ± 386	47684 ± 286	H = 3.83, *p* = 0.14; n.s.
Mn^2+^	0.07 ± 0.06	0.094 ± 0.018	0.093 ± 0.007	H = 0.12, *p* = 0.85; n.s.
Ge	0.028 ± 0.0011	0.029 ± 0.0006	0.03 ± 0.0007	H = 0.94, *p* = 0.62; n.s.
I	1.03 ± 0.24	0.75 ± 0.16	0.87 ± 0.23	H = 1.44, *p* = 0.48; n.s.
P	189 ± 7.26	182.2 ± 7.03	179 ± 4.61	H = 79, *p* = 0.5; n.s.

* *p* < 0.05 vs. Control ^#^ *p* < 0.05 vs. Amalgam.

**Table 4:** Heavy metals of environmental exposure among experimental groups.

Metal (µg/g Hair)	Control (Cont)	Amalgams (A)	Implants + Amalgams (A + I) H (A + I, KW)	*p*-Value
Ba	0.28 ± 0.04	0.57 ± 0.13 *	0.92 ± 0.13 *^,#^	H = 15.77, *p* < 0.05
Pb	0.2 ± 0.066	0.29 ± 0.063	0.7 ± 0.23	H = 1.07, *p* = 0.58; n.s.
Cd	0.012 ± 0.0025	0.009 ± 0.0004	0.026 ± 0.015	H = 3.18, *p* = 0.2; n.s.
Sb	0.01 ± 0.0046	0.012 ± 0.0027	0.013 ± 0.001	H = 0.81, *p* = 0.6; n.s.
As	0.030 ± 0.0028	0.031 ± 0.0026	0.029 ± 0.004	H = 1.6, *p* = 0.33; n.s.
Pt	0.06 ± 0.0091	0.064 ± 0.0077	0.068 ± 0.0026	*p* > 0.05, n.s.
Tl	0.0010 ± 0.0006	0.0010 ± 0.0004	0.0011 ± 0.0006	*p* > 0.05, n.s.
Th	0.0011 ± 0.0045	0.0010 ± 0.0085	0.0011 ± 0.005	*p* > 0.05, n.s.
U	0.001 ± 0.0004	0.0011 ± 0.0008	0.0011 ± 0.0008	*p* > 0.05, n.s.

* *p* < 0.05 vs. Control ^#^ *p* < 0.05 vs. Amalgam.

**Table 5:** Correlations between Mo/Hg^2+^, Mo/Co, Mo/Fe^2+^ ratios in the A + I group by r Pearson or r Spearman.

Correlations r Pearson/Spearman *p*-Value	Mo/Co	Mo/Fe^2+^
**Mo/Hg^2+^ (A + I)**	*r* = 0.45 *p* = 0.005	*r* = 0.62 *p* = 0.000048
	**Ca^++^**	**Fe^2+^**
**Mo (A + I)**	*r* = −0.37 *p* = 0.034	*r* = −0.4 *p* = 0.025
	**Mn++**	**Cr**
**Hg^2+^ (A + I)**	*r* = −0.34 *p* = 0.048	*r* = 0.49 *p* = 0.016
	**Cr**	**Cu^2+^**
**V**	*r* = 0.56 *p* = 0.004	*r* = 0.49 *p* = 0.0034
